# Community-led trials: Intervention co-design in a cluster randomised controlled trial

**DOI:** 10.1186/s12889-017-4288-6

**Published:** 2017-05-30

**Authors:** Neil Andersson

**Affiliations:** 1 Centro de Investigación de Enfermedades Tropicales (CIET), Universidad Autónoma de Guerrero, Acapulco, Guerrero Mexico; 2 Department of Family Medicine, McGill University, Montreal, Canada

## Abstract

In conventional randomised controlled trials (RCTs), researchers design the interventions. In the Camino Verde trial, each intervention community designed its own programmes to prevent dengue. Instead of fixed actions or menus of activities to choose from, the trial randomised clusters to a participatory research protocol that began with sharing and discussing evidence from a local survey, going on to local authorship of the action plan for vector control.

Adding equitable stakeholder engagement to RCT infrastructure anchors the research culturally, making it more meaningful to stakeholders. Replicability in other conditions is straightforward, since all intervention clusters used the same engagement protocol to discuss and to mobilize for dengue prevention. The ethical codes associated with RCTs play out differently in community-led pragmatic trials, where communities essentially choose what they want to do. Several discussion groups in each intervention community produced multiple plans for prevention, recognising different time lines. Some chose fast turnarounds, like elimination of breeding sites, and some chose longer term actions like garbage disposal and improving water supplies.

A big part of the skill set for community-led trials is being able to stand back and simply support communities in what they want to do and how they want to do it, something that does not come naturally to many vector control programs or to RCT researchers. Unexpected negative outcomes can come from the turbulence implicit in participatory research. One example was the gender dynamic in the Mexican arm of the Camino Verde trial. Strong involvement of women in dengue control activities seems to have discouraged men in settings where activity in public spaces or outside of the home would ordinarily be considered a “male competence”.

Community-led trials address the tension between one-size-fits-all programme interventions and local needs. Whatever the conventional wisdom about how prevention works at a system level, programmes have to be perceived as locally relevant and they must engage stakeholders who make them work. Locally, each participating community has to know the intervention is relevant to them; they have to want to do it. That happens much more easily if they design the programme themselves.

## Article

The British Medical Journal published the multi-centred *Camino Verde* cluster randomised controlled trial [1] as the first report of a community mobilisation intervention leading to serological evidence of reduced dengue virus infection. Beyond dengue prevention, publication of the *Camino Verde* trial in the BMJ showed that community-led trials can be reported in high quality journals.

In conventional randomised controlled trials (RCTs), researchers design the interventions. In the RCT world of researcher hypotheses, concepts like fidelity and reproducibility have been interpreted to mean that all participants – all clusters in a cluster RCT – must receive verifiably the same intervention. In the *Camino Verde* trial, each intervention community designed its own set of actions to prevent dengue. Instead of a fixed prevention programme or menu of activities to choose from, the trial randomised clusters to a *participatory research protocol* that began with sharing and discussing evidence, leading to local authorship of the action plan for vector control. A similar protocol, without the vector-specific references, might be applicable to many health or social issues.

### Evolution of RCT methods

Public health research has evolved beyond observational studies, increasingly to include RCTs. These experiments involve comparisons with people who do not receive the intervention (controls), and rely on random allocation of the intervention to generate convincing evidence of impact or lack of impact. The consequence of randomisation is that exposure to the intervention is independent of all events and relationships that precede it – it converts potential confounders and other covariates into random differences. This lifts the onerous and often futile burden of proof of observational studies, where the researcher is obliged to exclude potential confounders as possible explanations of a presumed effect [2, 3].

In the three-quarters of a century since the first published RCT in the 1930s [4], we have seen major developments of trial methods in several directions. The biggest volume of health-related RCTs is undisputedly the clinical trials sponsored by the pharmaceutical industry [5], essentially to comply with legal requirements about proof of efficacy. There has also been a surge of the n-of-1 clinical management trials used in personalised medicine [6, 7] and most recently the emergence of pragmatic trials with implications for large scale public health policy [8–10].

Pragmatic trials refocus RCT methodology such that randomising and comparing with a control group are the principal contributions of the trial; there is no longer any double blinding or placebos that were almost synonymous with efficacy trials [11]. The related surge in public policy trials brought to light the advantage of clusters to reduce contamination bias for educational and mobilisation interventions; where interventions work *between people*, instead of trying to isolate people to avoid spill-over, it makes sense to intervene at cluster level.

There have also been shifts in the definition of the intervention in trials. Brown and others [12] defined adaptive trials as having planned modification of characteristics of the intervention based on information from the accumulating data. Product development adaptive trials, borrowed from business studies, now talk about involving “consumers” in programme development at all stages [13, 14]. Community-led trials, although with very different origins in participatory research, benefit from some of these technical developments in RCT methods.

### Participatory research in an epidemiological framework

Participatory research is an umbrella term that includes partnered research, community-based participatory research, action research, participatory action research, participatory evaluation, community and patient engagement [15]. It implies the systematic co-creation of new knowledge in equitable partnerships with people affected or those who will benefit from or act on it [16, 17]. Although participatory research for some people implies small scale, very local exercises, there is nothing in its definition that excludes national and multi-national participatory research operations [18].

Adding equitable stakeholder engagement to RCT infrastructure has several benefits [19]: cultural anchoring shapes the scope and direction of research, making it more meaningful to stakeholders; recruitment of community members to management roles builds capacity; the dialogic approach leads to creative turbulence that, when resolved, promotes positive and sustained outcomes; success increases confidence over time, with implications for other health issues; goals are often sustained beyond funded time frames; and systemic changes achieved from the engagement have wider implications.

An early example of a community-led trial, *Rebuilding from Resilience* [20]*,* was a partnership of 12 Indigenous women’s shelters across Canada. This tested the impact and cost implications of evidence-based community-led initiatives to decrease domestic violence. The women’s shelter directors took the driving seat in their own research. They viewed randomisation as the only fair way to decide whose turn it was to receive the available resources; each shelter director drew a number out of a hat, indicating whether their shelter would join the first wave or the second wave. In each community, a detailed development and consultation process led to a baseline study, using other gender violence questionnaires as reference. The results fuelled a series of discussions and workshops on how to prevent gender violence. The baseline for the second wave provided the unexposed contrast for the follow-up study of the first wave, after 2 years of interventions.

### Implementation and interpretation of community-led trials

*Camino Verde* illustrates some issues in the design, implementation and interpretation of community-led trials.

#### Replication of the intervention

Because *Camino Verde* randomised a participatory research protocol, the conclusion of the trial should strictly be that participatory research – rather than specific vector control actions – added value to existing programmes attempting to reduce dengue virus infection. There is no problem about ensuring replication [21] in other conditions, since each intervention cluster received the same evidence-based engagement protocol to discuss and to mobilise for dengue prevention [22].

#### Ethics

The stringent ethical codes associated with RCTs, including informed consent [23], play out differently in explanatory trials and community-led pragmatic trials, where communities essentially choose what they want to do [24]. Issues of withholding interventions in control communities [25] can be settled by randomising the delay among all eligible communities, as in a stepped wedge design. Residual problems include demonstrating respect for community autonomy when the locus of research shifts from the individual to community level [26].

#### Intervention co-design

Intervention co-design is neither easy nor automatic, and communities do not always come up quickly with the most effective solutions. In *Camino Verde*, the approach to community-led design took 3 years to develop prior to the main trial. Once the protocol was in place, many residents started with the idea that government should solve their dengue problem. Between the different discussion groups in each intervention community that led to multiple plans for prevention, most communities developed their compound intervention recognising different time lines; they chose some fast turnarounds, like elimination of breeding sites, and longer term actions like garbage disposal and improving water supplies.

#### Biases

Selection bias can be reduced by concealment of centralised allocation of the intervention. As in most pragmatic trials, blinding and placebos are impossible in community-led trials, and biases can result from service providers and communities knowing what the interventions are. In a community-led trial a Hawthorne effect (an impact because the group receiving the intervention know they are receiving an intervention) is likely and is part of the intervention benefit. Practical steps to ensure knowledge of allocation of the intervention affects the outcome measure as little as possible can include using biological outcomes (such as serological evidence of dengue infection in the *Camino Verde* trial) rather than responses from officials. In interventions where participants choose what they do, it is possible that their choice of intervention is influenced by what they read [27]. In *Camino Verde,* communities chose the intervention based on evidence of local vector habits and community discussions, so there was little risk of a publication bias.

#### Trial implementation

In any trial, a rigid and well documented protocol helps avoid undisclosed implementation flexibility [28]; this is also true for community-led trials, when the intervention is to share information and co-design solutions. What individual intervention communities opted to do in the *Camino Verde* trial was up to them, but the work of the *Camino Verde* trial team adhered tightly to the protocol. Mostly this involved training of facilitators and handing over the evidence for discussion.

#### Analysis

In any cluster trial, a lot of analytic power is foregone when cluster is the unit of randomisation and the unit of intervention [29]. The *Camino Verde* main analysis per protocol was the most conservative possible for a cluster trial: a t-test treated each cluster as a unit and the outcome rate as continuous variables in each cluster. Strictly following protocol, as happened in the *Camino Verde* analysis, avoids *p*-hacking [30] and hypothesizing after the results are known (HARKing) [31]. This is especially important in community-led trials where local initiatives can give rise to unexpected and interesting ways of doing things. A *Camino Verde* example was the reintroduction of larvivorous fish in some communities; this generated interesting supplementary results [32] but did not influence the trial’s principal analysis.

#### Ambiguity of indicators

Ambiguity of indicators and difficulty in defining the measurement parameters are not avoided by randomisation and can cause problems in clinical, pragmatic and community-led trials. With the further variability introduced by different packages of solutions in each participating community, a reliable endpoint is helpful. *Camino Verde* opted for a hard biological endpoint: serological evidence of dengue virus infection.

#### Skills and infrastructure

Introduction of high level research methods and participatory research into local programme development has multiple advantages, including improving the programme in question, but it requires a quantum shift in skills and sensibilities. The key to almost any trial is in the measurement skill. In community-led trials, additional skills of promoting dialogue, often in an intercultural context, are indispensable. An article by Morales-Pérez and colleagues describes the training of Mexican facilitators in the *Camino Verde* trial [33]. A big part of the skill set is being able to stand back and simply support communities in what they want to do and how they want to do it, something that does not come naturally to many vector control programs.

#### Unexpected negative outcomes

The turbulence implicit in any participatory research process can in the short term introduce and exacerbate local frictions. One example of this is the gender dynamic that seems to have resulted from the surge of interest and involvement of women and children in the Mexican arm of the *Camino Verde* trial [34]. Analysis indicated interruptions of the results chain between knowledge and preventive action in men exposed to the intervention. It seems plausible that the strong involvement of women in dengue control activities had a negative effect on the men in communities where activity in public spaces or outside of the home would ordinarily be considered a “male competence”.

### The way forward

The *Camino Verde* trial is an interesting precedent for community-led RCTs in public health, addressing the well-known tension between programme interventions and local needs. For logistical and administrative purposes, public health programmes have objectives, actions to reach those objectives, outputs, outcomes and impacts. Unfortunately, that strong sense of system does little to make programmes locally relevant or engaging to stakeholders who must make them work. Locally, each participating community has to *know* the intervention is relevant to them; they have to *want* to do it. That happens much more easily if they design the programme themselves.

Public health practitioners are increasingly recognising that stakeholder engagement and participation is crucial for success. There is nothing new in this proposition, being a founding principle of the 1978 primary health care concept at Alma Ata [35]. The decentralised nature of engagement and participation has long been held to be contradictory to any one-size-fits-all formulation. Community-led RCTs provide informative answers to a few very specific questions.

Where the objective of the exercise is very clear – in this case it was to control *Aedes aegypti –* and there are several options of how to achieve this, quite what communities do should not matter so much as that they do get involved.
